# *Populus trichocarpa PtNF-YA9*, A Multifunctional Transcription Factor, Regulates Seed Germination, Abiotic Stress, Plant Growth and Development in *Arabidopsis*

**DOI:** 10.3389/fpls.2018.00954

**Published:** 2018-07-09

**Authors:** Conglong Lian, Qing Li, Kun Yao, Ying Zhang, Sen Meng, Weilun Yin, Xinli Xia

**Affiliations:** ^1^Beijing Advanced Innovation Center for Tree Breeding by Molecular Design, Beijing Forestry University, Beijing, China; ^2^National Engineering Laboratory for Tree Breeding, Beijing Forestry University, Beijing, China; ^3^College of Biological Sciences and Technology, Beijing Forestry University, Beijing, China; ^4^Key Laboratory of Genetics and Breeding in Forest Trees and Ornamental Plants, Beijing Forestry University, Beijing, China

**Keywords:** *Populus trichocarpa*, *NF-YA*, post-germination growth arrest, drought tolerance, plant development

## Abstract

*NF-YAs* play important roles in abiotic stress. However, their characteristics and functions in abiotic stress of *poplar*, a model woody plant, have not been fully investigated. Here, the biological functions of *PtNF-YA9* (Potri.011G101000), an *NF-YA* gene from *Populus trichocarpa*, were first fully investigated. PtNF-YA9 is located in the nucleus. The expression of *PtNF-YA9* was reduced by mannitol, NaCl, and abscisic acid (ABA). The GUS staining of ProNF-YA9::GUS transgenic lines was also reduced by mannitol treatments. In the *PtNF-YA9*-overexpressed *Arabidopsis* (OxPtNA9), OxPtNA9 lines exhibited sensitivity to simulated drought, ABA, and salinity stress during germination stage, and growth arrest emerged at post-germination stage. These phenomena might involve the ABA signaling pathway via the regulation of *ABI3*, *ABI4*, and *ABI5*. At vegetative stages, OxPtNA9 lines decreased in water loss via promoting stomatal closure and displayed high instantaneous water-use efficiency (WUE) of the leaf to exhibit enhanced drought tolerance. Furthermore, OxPtNA9 lines exhibited long primary root in the half-strength Murashige–Skoog agar medium supplemented with NaCl and conferred strong tolerance in the soil under salt stress. Additionally, *PtNF-YA9* exhibited dwarf phenotype, short hypocotyl, small leaf area and biomass, delayed flowering, and increased chlorophyll content. Above all, our research proposes a model in which *PtNF-YA9* not only plays a key role in reducing plant growth but also can play a primary role in the mechanism of an acclimatization strategy in response to adverse environmental conditions.

## Introduction

Plants are usually subjected to various abiotic challenges from the environment, especially in extreme temperature, high salinity, and long-term drought. Drought is a major disadvantageous environmental factor limiting plant development and growth; it is widespread in many regions and is expected to progressively increase (Burke et al., 2006). With the development of biotechnology, the identification and application of genetic transformation technology to enhance stress tolerance of plants are essential for screening and breeding new resistant plants (Valliyodan and Nguyen, 2006). In recent decades, the complicated signaling network response to different abiotic stresses has been relatively and thoroughly defined and characterized. In terms of gene regulation, transcription factor families (Zhu, 2002), including *NAC* (Lu et al., 2017), *MYB* (Wei et al., 2017), *AP2*/*ERF* (Ahn et al., 2017), *bHLH* (Dong et al., 2014), and *WRKY* (Jiang et al., 2014), all play significant roles in regulating the expression level of functional genes to enhance drought tolerance. Thus, many studies have investigated the identification and application of transcription factors in plant genetic engineering to increase plant resistance.

NUCLEAR FACTOR Y (NF-Y) transcription factor, also called the Heme Activator Protein (HAP) or CCAAT-binding factor (CBF), is pervasive in high eukaryotes. The *NF-Y* member is deemed as a heterotrimeric transcription factor and includes three subunits, namely, *NF-YA* (*HAP2* or *CBF-B*), *NF-YB* (*HAP3* or *CBF-A*), and *NF-YC* (*HAP5* or *CBF-C*) (Forsburg and Guarente, 1989). The heterodimers of NF-YB/NF-YC were supposed to translocate into the nucleus from the cytoplasm and then interact with *NF-YA* in the nucleus to form heterotrimers (Frontini et al., 2004; Steidl et al., 2004). *NF-Ys* participate in a series of biological processes, such as flower development (Cao et al., 2014), primary and secondary metabolism (Li et al., 2015), embryo and seed development (Lee et al., 2003), root development (Sorin et al., 2014), and nutrition balance (Pant et al., 2009), and they are also involved in multiple abiotic stresses (Leyva-González et al., 2012; Han et al., 2013). Notably, *NF-YA* transcription factors, have been shown to be crucial for responses to plant abiotic stress. For instance, *AtNFYA5* confers drought resistance via transcriptional and posttranscriptional regulation (Li et al., 2008). *AtNF-YA2*, *AtNF-YA*7, and *AtNF-YA10* overexpressing lines all result in dwarf phenotypes and confer several types of abiotic stress tolerance (Leyva-González et al., 2012). Furthermore, overexpression of *OsHAP2E* gene increases multifunction resistance, such as pathogen tolerance and resistance to high drought and salinity in rice (Alam et al., 2015). Moreover, some *NF-YA* genes negatively regulate several types of stress tolerance. For instance, wheat *TaNF-YA10* increases sensitivity to salinity in *Arabidopsis thaliana* (Ma et al., 2015). In addition, *NF-YAs* also play a distinct role under different stresses. For instance, overexpression of *GmNFYA3* confers enhanced drought resistance but exhibits sensitivity to high salt stress (Ni et al., 2013). Thus, it is extremely rewarding to screen and study the functions of members in the *NF-Y* transcription factor family.

*Populus trichocarpa* has a modest genome size and acts as a model for the study of woody plants (Tuskan et al., 2006). Only a few *NF-Y* genes from woody plants have been functionally characterized, especially in poplar. *PtrHAP2* is involved in vegetative bud dormancy in *Populus* (Potkar et al., 2013), whereas *PagHAP2-6* is involved in poplar cambium dormancy and the regulation of ABA (Ding et al., 2016). Our previous studies on *NF-YB7* showed that *PdNF-YB7* overexpression increases *Arabidopsis* water-use efficiency (WUE) and drought resistance (Han et al., 2013). Meanwhile, the function of poplar *NF-YA* in abiotic stresses has not yet been studied. The expression of *PtNF-YA9* is reduced under drought condition based on RNAseq data from Popgenie^1^, but its *Arabidopsis* homolog *AtNF-YA7* confers different abiotic stress tolerance (Leyva-González et al., 2012). These findings pose an interesting question on how this gene acts under drought condition in *Populus*. To answer this question, quantitative reverse transcription polymerase chain reaction (RT-qPCR) was used, and overexpression of *PtNF-YA9* transgenic *Arabidopsis* lines was generated for function analysis. Here, multifunctional phenotypes of *PtNF-YA9* were observed, and the drought tolerance and possible function mechanisms of *PtNF-YA9* were further investigated.

## Materials and Methods

### Plant Materials and Growth Conditions

In this study, wild-type (WT) Col-0 of *A. thaliana* and mutant line *nfya7* (SALK_121158.47.00.x) on the Col-0 background were used. Seeds of different *Arabidopsis* lines were sterilized with ethanol (75%) for 1 min followed by NaClO (1%) for 10 min. The seeds were subsequently washed with distilled water for four times before sowing. After sowing, *Arabidopsis* seeds were kept at 4°C for 48 h for vernalization. The seeds, except those used in germination tests, were grown on half-strength Murashige–Skoog (1/2 MS) medium containing 2% sucrose (0.6% agar, pH 5.8) in a plant growth chamber (22°C) under 16 h light photoperiods of white light (120 μmol m^-2^ s^-1^). After germination, 12-day-old *Arabidopsis* seedlings were transplanted to a pot with a mixture of turfy soil, perlite, and vermiculite (2:1:1) and grown in an illumination incubator with relative humidity of 70% under a 16 h/8 h light/dark photoperiod (120 μmol m^-2^ s^-1^) at 23°C.

*Populus trichocarpa* plantlets were preserved in the Beijing Forestry University by tissue culture. *P. trichocarpa* mature plants were obtained through 3 months of natural growth after transplanting from *in vitro* plantlet. The plantlets were transplanted in 4 L pots with a mixture of loam. Hoagland nutrient solutions were used to water every 2 weeks in the greenhouse for 3 months until the experiments. For dehydration stress treatment, uniformly grown *P. trichocarpa* plants were washed from pots, and natural dehydration was perfomed at similar conditions of room temperature. Moreover, between six and eight nodes of leaves were harvested at four time points of 0, 0.5, 1, and 4 h. For salt stress treatment, uniformly developed *P. trichocarpa* plants were fully watered using 200 mM NaCl solution. For ABA treatment, 300 μmol ABA was evenly sprayed on the leaves of poplar. The leaves between six and eight nodes of NaCl and ABA treatments were harvested at four time points at 0, 1, 4, and 8 h. All harvested samples were frozen in liquid nitrogen immediately and then preserved at -80°C for later use.

### Cloning and Sequence Analysis of *PtNF-YA9* Gene

To obtain the cDNA sequence of *PtNF-YA9*, total RNA was extracted from *P. trichocarpa* by using the EASYspin Plus Plant RNA Kit (AidLab, Beijing, China) following the manufacturer’s instructions. Then, cDNA was synthesized using the TIANGEN FastQuant RT Kit (Qiagen, Hilden, Germany). The specific primers NA9-F and NA9-R (**Supplementary Table S1**) were designed according to the full-length cDNA reference sequence obtained from PopGenIE, and the open reading frame (ORF) sequence of *PtNF-YA9* (Potri.011G101000) was amplified from the cDNA of *P. trichocarpa* via PCR and then cloned into the pMD18-T vector. The new vector was named as PtNA9-T. The functional region of *PtNF-YA9* was analyzed by InterPro^2^. Physical and chemical parameters were analyzed by ExPASy^3^. Multiple sequence alignment of amino acid sequences was analyzed using ClustalW. Phylogenetic trees between *P. trichocarpa NF-YA* homology proteins were constructed using MEGA software based on neighbor-joining method with 1000 bootstrap replications.

### Subcellular Location of the PtNFYA9-GFP Fusion Protein

The full-length coding sequence (CDS) of *PtNF-YA9* without the stop codon was amplified from PtNA9-T by PCR using gene-specific primers NA9-GFP-Fz and NA9-GFP-Rz (**Supplementary Table S1**). For the expression of the 35S::PtNF-YA9-GFP fusion protein, the PCR product was cloned between the Cauliflower mosaic virus (CaMV) 35S promoter and GFP gene in the pCambia1304 vector to form NF-YA9 and GFP fusion protein. The construct fusion vector was confirmed by sequencing and then transformed into *Agrobacterium* strain *LBA4404* and subsequently infiltrated into the *Nicotiana benthamiana* leaves for transient expression via *Agrobacterium*-mediated gene transformation (Li, 2011). The 3 days *N. benthamiana*-transformed leaves after infiltration were observed under a confocal microscope (Nikon).

### Expression Analysis of *PtNF-YA9* in *P. trichocarpa*

To examine the expression levels of *PtNF-YA9* under different stresses and tissues, real-time qPCR was performed using primers NA7-qF and NA7-qR (**Supplementary Table S1**) by SuperReal PreMix Plus (SYBR Green) (TIANGEN, Beijing, China). The procedure followed the manufacturer’s instructions, the relative quantification value was calculated using the 2^-ΔΔCt^ method, and the kinetics of PCR product was monitored using SYBR Green (Steibel et al., 2005). The transcript levels of *GAPDH* (AT1G16300) or *Actin2* (AT3G18780) were used to quantify the expression of detected genes in samples. RT-PCR was also carried out to investigate the expression of *PtNF-YA9* in transgenic *Arabidopsis* lines. PCR amplification (94°C for 30 s, 56 °C for 30 s, and 72°C for 1 min) was performed for 40 cycles, and each PCR assay was replicated for three biological replicates. All the primers used for RT-PCR and RT-qPCR are shown in **Supplementary Table S1**.

### Promoter Isolation, *cis*-Acting Element, and GUS Staining Analysis

To isolate the promoter sequence of *PtNF-YA9*, DNA was extracted from *P. trichocarpa* by CTAB method, and the reference genomic DNA sequence of *PtNF-YA9* was searched in Phytozome. Based on the sequence identified from *Populus trichocarpa* v3.0 database, gene-specific primers NA9Pro-F and NA9Pro-R (**Supplementary Table S1**) were designed, and the sequence was amplified by a PCR cover at approximately 2000 bp upstream of the start codon. PlantCARE online database was used to predict *cis*-acting regulatory elements (Lescot et al., 2002). The *PtNF-YA9* promoter adapter sequence was obtained by gene-specific primers NA9Pro-Fz and NA9Pro-Rz (**Supplementary Table S1**) and was then cloned into pCambia1301 vector instead of *CaMV 35S* promoter with the Seamless Assembly Cloning Kit (CloneSmarter). The constructed vector was sequenced and then transferred into *Agrobacterium* strain *GV3101*. The transgenic *Arabidopsis* plants were obtained by the floral dip method (Clough and Bent, 1998). To detect the promoter of GUS staining, 16-day-old transgenic *Arabidopsis* seedlings grown on 1/2 MS agar medium plates were transplanted into 1/2 MS agar medium plates with or without 200 and 250 mM mannitol for 24 h.

### Constructs and Generation of the *PtNF-YA9* Transgenic *Arabidopsis* Plants

The ORF sequence of *PtNF-YA9* was obtained from PtNA9-T vector using the specific primers NA7-Fz and NA7-Rz by PCR (**Supplementary Table S1**). The PCR product was cloned into the pCambia1301 vector containing the *CaMV 35S* promoter instead of *GUS* gene with the Seamless Assembly Cloning Kit (CloneSmarter). The constructed vector was sequenced and then transferred into *Agrobacterium* strain *GV3101*. The *Arabidopsis* plants were also transformed by the floral dip method (Clough and Bent, 1998).

### Germination Assays and Cotyledon Greening Rate Analysis

For the germination assay, three replicates of 50 seeds from different lines of *Arabidopsis* were surface-sterilized. The surface-sterilized seeds were sown on 1/2 MS agar medium supplemented with mannitol, NaCl, or ABA. The germinated seeds were counted based on the radicles protruding from the seed coat and statistically analyzed every day until 11 days. Moreover, cotyledon greening rate was analyzed when the germinated seeds established seedlings and cotyledon turned green.

### Drought and Salt Experiments at Seedling and Growth Stages

For the experiment of stress treatment at seedling stage, 4-day-old seedlings on 1/2 MS agar medium were transplanted to 1/2 MS agar medium plates supplemented with 200 mM mannitol and 100 mM NaCl. The growth phenotypes were compared, and the length of the primary roots was calculated. For long-term salt stress, 2-week-old *nfya7*, WT, OxPtNA9/nfya7, and OxPtNA9 lines were subjected to salt stress by pouring saltwater every 5 days until significant difference phenotypes were achieved, and the different phenotypes were photographed and recorded. For long-term drought treatment, the seedlings were watered for 2 weeks after transplanting into the pot. Then, the water was withheld until the difference phenotypes were achieved. Moreover, the differences in plant phenotypes were analyzed and photographed. Subsequently, the pots were re-watered and recovered for 3 days. With water deficit for 7 days, the photosynthetic indexes, which contain net CO_2_ assimilation, transpiration, and stomatal conductance, were measured by using the LI-6400 photosynthesis system (LI-COR 6400, Lincoln, NE, United States). Instantaneous leaf WUE was calculated as the ratio of net CO_2_ assimilation/transpiration. The leaf relative water content (RWC) was calculated as (FW - DW)/(TW - DW) × 100, which was described by Sade et al. (2014). The leaf chlorophyll content was extracted by 80% acetone, and the absorbance of extracting solution was measured at 663 and 645 nm. The chlorophyll a (Ca) content was calculated by Ca = 12.7 × A663 - 2.69 × A645, the chlorophyll b (Cb) content was calculated by Cb = 22.9 × A645 - 4.64 × A663, and the total chlorophyll content was defined as the sum of Ca + Cb.

### Water Loss Analysis and Stomatal Aperture Measurement

Detached rosette leaves of different lines grown for 3 weeks after transplanting into soil were weighed immediately and incubated on a white paper at room temperature. Losses of fresh weight in leaves were monitored at different time points of 0, 0.5, 1, 1.5, 2, 3, 5, and 8 h. Water loss is expressed as the percentage of initial fresh weight. To analyze the stomatal apertures, the leaves were incubated in a solution containing 50 mM KCl, 50 mM CaCl_2_, and 10 mM MES/KOH (pH 6.1) for 2 h under light condition. Final concentration of 30 μM ABA was then added. Stomatal apertures were observed with a microscope and measured using Photoshop software after 1 h of ABA treatment. For each experimental repeat, at least 50 stomata were calculated and measured. The values of stomatal width to length ratios acted as the indicator of stomatal phase and were divided into three phases. The stomata was defined as open when the ratio was greater than 0.5 μm, partially closed between 0.5 and 0.2 μm, and closed with less than 0.2 μm.

### Statistical Analysis

All data were subjected to SPSS Statistics and Excel for analysis. Student’s *t*-test was used to detect the significant differences between individual means. Differences at the 1% level were considered significant and denoted by lowercase letters or asterisk among different groups.

## Results

### Cloning and Sequence Analysis of *PtNF-YA9*

A significant portion of the members were found to be involved in various abiotic stresses based on the systematic analysis of the *NF-Y* family in *P. trichocarpa*. Thirteen members of the *NF-YA* subfamily were identified in *P. trichocarpa* from Popgenie^4^ and PlantTFDB (Jin et al., 2017). Moreover, 11 members of *HAP2*/*NF-YA* genes have been already named in *P. trichocarpa* (Potkar et al., 2013). We added the remaining two members and used the same gene names in the study to avoid nomenclature confusion. One of them, designated as *PtNF-YA9*, the closest of *Arabidopsis* homolog *AtNF-YA7* with acclimatization strategy for abiotic stress tolerance, was selected for further functional characterization in this study.

**FIGURE 1:**
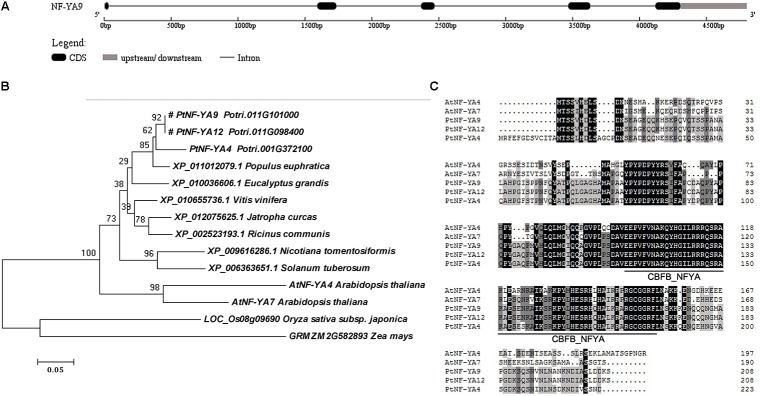
Gene structure, phylogenetic tree, and multiple sequence alignment of the PtNF-YA9 homology proteins across different plant species. **(A)** Gene structure diagram of PtNF-YA9. **(B)** Phylogenetic analysis of NF-YA proteins across different plant species. The amino acid sequences were aligned using MAFFT, and the phylogenetic tree was constructed using MEGA 6.0 software by neighbor-joining method with bootstrap analysis of 1000 replicates. The “#” label indicates target protein. **(C)** Multiple sequence alignment of PtNF-YA4, PtNF-YA9, PtNF-YA12, AtNF-YA4, and AtNF-YA7 proteins.

The cDNA sequence of *PtNF-YA9* (Potri.011G101000) was identified from the genome of *P. trichocarpa*. A 648-bp sequence containing a 627-bp CDS in length was obtained by specific primers of NA9-F and NA9-R. *PtNF-YA9* was predicted to encode 208 amino acids with a pI of 9.17, molecular mass of 22.76 kDa, and an instability index of 66.41 and is deemed as an unstable protein in nature. Moreover, the atomic composition forms a formula of C_986_H_1541_N_301_O_308_S_7_. The genomic sequence of *PtNF-YA9* was 4801 bp, including four introns and five exons (**Figure 1A**).

Phylogenetic tree of the PtNF-YA9 homology proteins across different plant species showed that these proteins were divided into two branches. The PtNF-YA9 of *P. trichocarpa* showed recent homology to *P. euphratica* XP_011012079 and *Eucalyptus grandis* XP_010036606 (**Figure 1B**). Multiple protein sequence alignment (**Figure 1C**) revealed that PtNF-YA9 and PtNF-YA12 proteins have the same amino acid sequence in the same chromosome, which is a phenomenon of gene duplication. Furthermore, they share the highest amino acid sequence homology to PtNF-YA4 and heterologous to AtNF-YA7 and AtNF-YA4 of *Arabidopsis* (**Figure 1C**). Phylogenetic tree also showed that PtNF-YA1, PtNF-YA3, and PtNF-YA10 are in the same small branch with PtNF-YA9 (**Supplementary Figure S1**). These proteins clustered in the same branch, indicating functional similarities and functional redundancy. These results generally indicated that PtNF-YA9 belongs to the NF-YA transcription factor family, which has a highly conserved CBFB_NFYA domain.

### Subcellular Localization of PtNF-YA9 Protein

**FIGURE 2:**
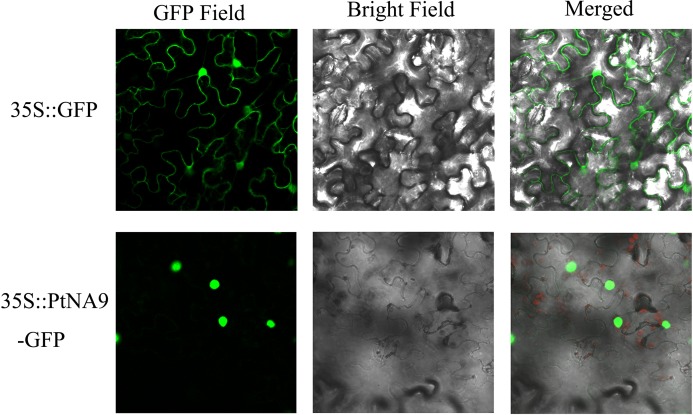
Subcellular localization of PtNF-YA9 protein. The 35S::GFP and 35S::PtNFYA9-GFP fusion protein transiently expressed in tobacco. Microscopic images contain green fluorescence, bright field, and merged microscope images.

Most transcription factors are localized in the nucleus. To determine the subcellular localization of PtNF-YA9 protein, the 35S:PtNFYA9-GFP and 35S:GFP vectors were constructed and introduced into epidermal cells of tobacco by using injection method. The fluorescence signals in the inner epidermal cells were observed using confocal laser scanning microscopy. PtNFYA9-GFP fusion protein was visualized in the nucleus, whereas the 35S:GFP (control) was observed throughout the cells (**Figure 2**). The results indicated that PtNF-YA9 is located in the nucleus.

### Expression Patterns of *PtNF-YA9* in *P. trichocarpa* in Response to Abiotic Stresses

**FIGURE 3:**
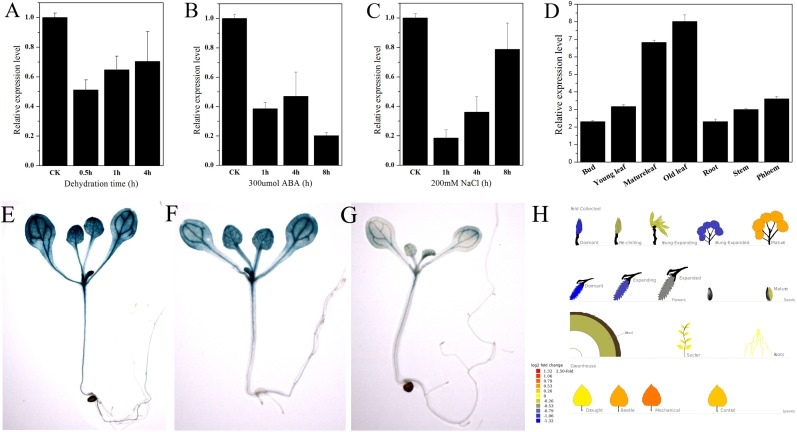
Expression profile of *P. trichocarpa NF-YA9*. **(A)** Expression pattern of *PtNF-YA9* in *P. trichocarpa* leaves under dehydration stress. **(B)** Expression pattern of *PtNF-YA*9 in *P. trichocarpa* leaves under 300 μM ABA. **(C)** Expression pattern of PtNF-YA9 in *P. trichocarpa* leaves under 200 mM NaCl. **(D)** Tissue expression pattern of *PtNF-YA9* in poplar. The expression levels were normalized to that of *UBQ*, and the level of *PtNF-YA9* transcript in the control was set at 1. Three biological repeats were performed, and each RT-qPCR was also performed thrice. **(E)** Seventeen-day-old ProNF-YA9::GUS transgenic *Arabidopsis* seedlings grown on 1/2 MS agar medium plates without treatment. **(F)** Sixteen-day-old ProNF-YA9::GUS transgenic *Arabidopsis* seedlings grown on 1/2 MS agar medium plates were transplanted into 1/2 MS agar medium with 200 mM mannitol for 24 h. **(G)** Sixteen-day-old ProNF-YA9::GUS transgenic *Arabidopsis* seedlings grown on 1/2 MS agar medium plates were transplanted into 1/2 MS agar medium with 250 mM mannitol for 24 h. **(H)** The expression patterns of *PtNF-YA9* in the Popgenie database (http://popgenie.org/gene?id=Potri.011G101000).

*PtNF-YA9* showed stress-response expression in the Popgenie database. The database showed that *PtNF-YA9* transcript was inhibited by drought stress and highly expressed in mature leaves than in the roots (**Figure 3H**). To further confirm the potential functions of *PtNF-YA9* in response to different abiotic stresses, the transcript abundance of *PtNF-YA9* in multiple organs and under a variety of abiotic stress treatments was detected by RT-qPCR. The results indicated that *PtNF-YA9* was actually inhibited by drought (**Figure 3A**). *PtNF-YA9* was also significantly downregulated by NaCl (**Figure 3C**) and ABA (**Figure 3B**), especially NaCl for 1 h and ABA for 8 h. These results indicated that *PtNF-YA9* might participate in the response of ABA, salt, and osmotic stresses.

We explored the expression pattern of *PtNF-YA9* at the transcriptional level regulated by abiotic stresses. The promoter of *PtNF-YA9* containing the 1985-bp genomic sequence upstream from their initiation codons was cloned, and *cis*-elements were analyzed (**Supplementary Figure S2**). There were some abiotic stress elements, such as HSE, MBS, TC-rich repeats, and WUN motif, and hormone response elements, such as CGTCA motif, TGACG motif, GA motif, and TCA-element. The prediction of possible transcription core promoter region sequence was AAGAACTTCAAA AAAATGCTGGTTTAGCCACATTTTGCTCATGCAAATGA from -808 bp to -658 bp by BDGP (**Supplementary Figure S2**). Furthermore, plant expression vector ProNF-YA9::GUS expressing the *GUS* gene under the control of the *PtNF-YA9* promoter was constructed, and the ProNF-YA9::GUS transgenic *Arabidopsis* lines were generated. GUS staining was used to detect the transcript abundance of the *GUS* reporter gene. As shown in **Figure 3**, the GUS signal was observed in almost all of the analyzed tissues/organs of seedlings, especially in leaf vasculature and shoot apical meristem (**Figure 3E**). A significant decrease of GUS staining of 16-day-old seedlings was observed for ProNF-YA9::GUS lines under 200 (**Figure 3F**) and 250 mM mannitol (**Figure 3G**) of 1/2 MS agar medium for 24 h. From the above findings, the expression patterns of ProNF-YA9::GUS were consistent with the results of RT-qPCR.

### Overexpression of *PtNF-YA9* Negatively Regulates Seed Germination and Leads to Post-germination Growth Arrest Under Abiotic Stress

**FIGURE 4:**
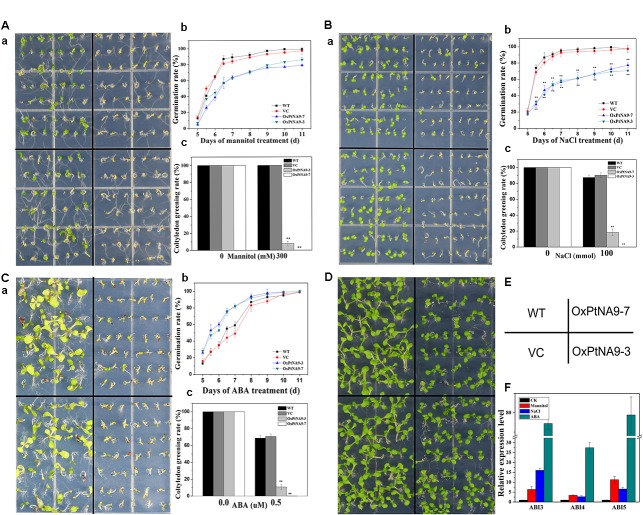
35S::PtNF-YA9 transgenic lines are hypersensitive to mannitol, salt stress, and ABA during germination growth. The 35S::PtNF-YA9 transgenic lines are hypersensitive to 300 mM mannitol **(A)**, 100 mM NaCl **(B)**, and 0.5 μM ABA **(C)** during germination stage. **(a)** Phenotypes of 35S::PtNF-YA9 transgenic lines are hypersensitive to mannitol, salt, and ABA. **(b)** Germination rate of different seeds on the mannitol, salt, and ABA medium counted for 11 days after sowing. **(c)** Cotyledon greening rate of different seeds on the mannitol, salt stress, and ABA medium after post-germination. **(D)** Control phenotype of 35S::PtNF-YA9 transgenic lines under 1/2 MS agar medium. **(E)** Sketch map of diagram a. **(F)** Accumulation of *ABI3* and *ABI5* in germinated seeds under mannitol, ABA, and salt treatment. The35S::PtNF-YA9 transgenic seeds were germinated on 1/2 MS liquid medium-moistened filter paper for 24 h after stratification and then were transferred onto filtered paper moistened with water (control), 300 mM mannitol, 100 mM NaCl, and 0.5 μM ABA. After treating for 8 h, the samples were harvested, and RNA was extracted. Here, three biological repeats were performed, and each RT-qPCR was also performed thrice.

To manifest the function of *PtNF-YA9* in the regulation of plant responses to abiotic stresses, overexpression of *PtNF-YA9* transgenic *Arabidopsis* (OxPtNA9) under the control of the *CaMV 35S* promoter was generated (**Supplementary Figure S3A**). Various expression levels of *PtNF-YA9* in several independent lines were obtained. OxPtNA9-7 and OxPtNA9-3 showed the highest mRNA levels by RT-PCR (**Supplementary Figure S3B**) and RT-qPCR (**Supplementary Figure S3C**) and were selected for further study. On the 1/2 MS agar medium, the seeds of OxPtNA9 lines exhibited slightly delayed germination than WT, and all of them showed good germination (**Figure 4D**).

The germination of progeny homozygous seeds were analyzed under osmotic stress. Less than 87% of *PtNF-YA9* transgenic seeds germinated in 300 mM mannitol-supplemented 1/2 MS agar medium after 11 days, whereas the germination of WT and VC exhibited more than 99 and 97%, respectively (**Figure 4A**). In addition, *PtNF-YA9* transgenic line OxPtNA9-7 failed to establish seedling, and OxPtNA9-3 showed less and smaller seedlings of approximately 7.69% compared with 100% seedlings of WT and VC (**Figure 4A**).

The seed germination and post-germination growth under salt stress was detected. As shown in **Figure 4B**, the OxPtNA9 germinated slowly, and the rates of germination were significantly reduced. Similarly, after seed germination, a notable post-germination growth arrest was also observed in OxPtNA9 in the 150 mM NaCl of 1/2 MS agar medium (**Figure 4B**). Then, OxPtNA9-7 germinated but could not develop into seedlings, and OxPtNA9-3 exhibited 18.52% smaller seedlings compared with 100% seedlings of WT and VC (**Figure 4B**).

Additionally, when we sowed the same WT and *PtNF-YA9* overexpressing lines on 1/2 MS agar medium supplemented with 0.5 μM ABA, the post-germination growth arrest was also exhibited, and a severe phenotype of arrest was found in the OxPtNA9 lines, establishing only 10.67% seedlings. Meanwhile, WT and VC can establish approximately 70% seedlings in the early stages (**Figure 4C**).

Furthermore, to determine the potential molecular mechanisms responsible for the post-germination growth arrest of *PtNF-YA9*, RT-qPCR was performed to detect the abundance of *ABI3*, *ABI4*, and *ABI5* at germination stage under different abiotic stresses. The results showed that the expression levels of *ABI3*, *ABI4*, and *ABI5* were significantly elevated under 300 mM mannitol, 100 mM NaCl, and 0.5 μM ABA at germination stage (**Figure 4F**). Overall, *PtNF-YA9* overexpression acted as a negative regulator of seed germination and led to post-germination growth arrest under different abiotic stresses possibly through *ABIs* of ABA signaling pathway.

### *PtNF-YA9* Confers High Drought Tolerance in Vegetative Growth Stage

**FIGURE 5:**
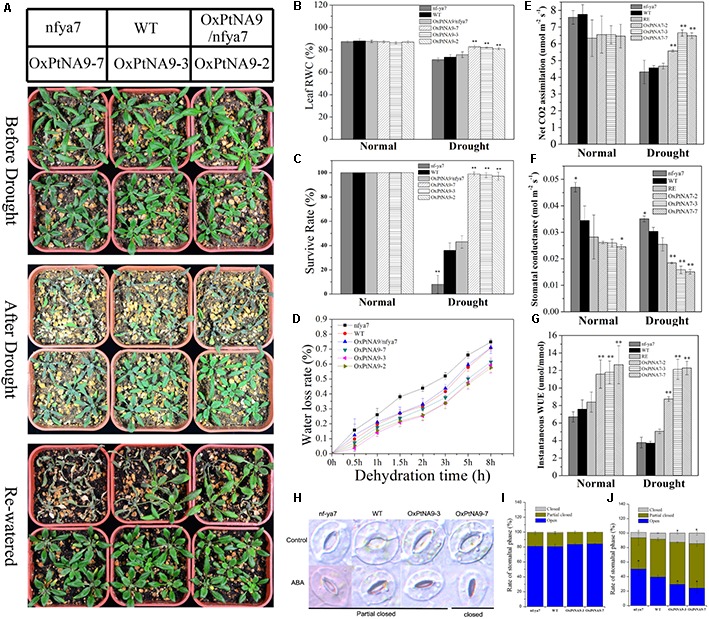
*PtNF-YA9* overexpression confers drought tolerance in *Arabidopsis*. **(A)** Morphological differences in drought experiments. **(B)** RWC of the leaves under normal and drought conditions. **(C)** Survival rate of seedlings under normal and drought conditions. **(D)** Water loss from detached leaves; water loss is expressed as the percentage of initial fresh weight of detached leaves. **(E)** Net photosynthetic rate. **(F)** Stomatal conductance. **(G)** Instantaneous WUE of different lines under normal and drought conditions. Under normal conditions, the data were collected on day 21 after being transferred to soil. Under drought conditions, the data were collected after 7 days of water withholding condition. All data are means from the six leaves for each of three independent experiments. **(H)** Typical phenotype of stomatal opening phase in transgenic lines, *nfya7*, and WT with or without ABA (30 μM) treatment for 2 h. **(I)** Percentages of the three types of stomata in transgenic lines, *nfya7* mutant, and WT plants are calculated in stomatal-induced liquid without ABA (30 μM) treatment for 1 h. **(J)** Percentages of the three types of stomata in transgenic lines, *nfya7* mutant, and WT plants are calculated with ABA (30 μM) treatment for 2 h.

*PtNF-YA9* transgenic seeds displayed a drought-sensitive phenotype (**Figure 4A**) and an ABA-hypersensitive phenotype (**Figure 4C**). OxPtNA9 was deemed as the resistance phenotype because of the small and green rosette leaves (**Figure 5**) (Achard et al., 2008). Hence, we investigated whether *PtNF-YA9* transgenic plants displayed an altered phenotype in response to drought stress (**Figure 5**). At seedling stages, 7-day-old seedlings of *nfya7*, WT, OxPtNA9/nfya7, and OxPtNA9 lines were transferred to 1/2 MS agar medium with or without 200 mM mannitol. Without 200 mM mannitol, the primary roots were shorter but showed no significant differences, whereas the lateral roots were significantly increased in the OxPtNA9 lines (**Supplementary Figure S4**). With 200 mM mannitol, the results indicated no significant differences in the length of the primary root, but OxPtNA9 lines showed more lateral roots than *nfya7* and WT (**Supplementary Figure S5**).

To examine the effects of *nfya7*, WT, OxPtNA9/nfya7, and OxPtNA9 lines on long-term drought condition, we subjected 2-week-old *nfya7*, WT, OxPtNA9/nfya7, and OxPtNA9 plants to water withholding conditions until significant phenotype appeared, and they were then re-watered. Under normal growth condition, smaller rosette phenotypes of OxPtNA9 transgenic lines were observed compared with WT plants (**Figure 5A**). However, with intensifying and lasting drought stress, *nfya7* mutant, WT, and OxPtNA9/nfya7 plants displayed a more withered phenotype than *PtNF-YA9* transgenic plants (**Figure 5A**). Photosynthesis analysis showed that *nfya7* mutant and WT were significantly reduced and maintained a significantly lower photosynthetic rate than OxPtNA9 transgenic lines under drought treatment (**Figure 5E**). Under normal growth conditions, the stomatal conductance of *nfya7* mutant was the highest, followed by the WT and then OxPtNA9 transgenic lines. Meanwhile, under drought condition, the stomatal conductance of OxPtNA9 lines was significantly lower than that of WT and *nfya7* mutant (**Figure 5F**). The transpiration rates of OxPtNA9 lines were also significantly lower than those of WT and *nfya7* mutant under both well-watered and drought conditions. These photosynthesis results showed a significant increase in the instantaneous leaf WUE of OxPtNA9 lines (**Figure 5G**). Furthermore, the OxPtNA9 lines had higher leaf RWC compared with WT and *nfya7* mutant under drought condition (**Figure 5B**). Thus, *PtNF-YA9* overexpression was demonstrated to improve water deficiency tolerance in *Arabidopsis* at the vegetative growth stage. In addition, OxPtNA9 lines recovered more quickly than WT plants after re-watering for 3 days (**Figure 5A**). The survival rate of OxPtNA9 lines was 97–100%. Meanwhile, the survival rates of WT and OxPtNA9/nfya7 were 36% ± 6.3% and 43% ± 5.2%, respectively. The *nfya7* mutant plants almost could not recover (**Figure 5C**).

To explore the physiological mechanism of the drought-resistance phenotype displayed by *PtNF-YA9*, a water loss assay with the fresh weight of detached rosette leaves by times as an indirect indication of the transpiration rate was performed. We discovered that the rate of water loss was lower in *PtNF-YA9* overexpressing lines than in WT. After dehydration treatment for 8 h, the fresh weight of WT decreased by 70.83% ± 1.85%, but that of the three OxPtNA9 lines only lost 59.28 ± 5.22%, 57.54 ± 3.15%, and 61.40 ± 1.81% (**Figure 5D**). This result indicated that the drought-tolerant phenotype of OxPtNA9 lines was attributed to enhance the water retention capacity.

In plants, water loss is regulated by guard cells, which cause the stomata to open and close. ABA as an exogenous hormone can induce stomatal closure. Studies have showed that *PtNF-YA9* is involved in ABA signaling. To further demonstrate whether *PtNF-YA9* participates in the regulation of stomatal aperture, the phenotypes of the stomata of *nfya7*, WT, and OxPtNA9 lines were compared. According to the stomatal aperture, we divided the stomatal types into three categories as open, partially closed, and closed (**Figure 5H**). After inducing full stomatal opening, the stomas in plants were primarily completely open and partially closed, and the rates of these three stomatal phases were similar among WT, *nfya7*, and OxPtNA9 lines (**Figure 5I**). However, after 2 h of ABA treatment, the rates of closed and partially closed stomata increased remarkably, especially in the OxPtNA9 lines. By contrast, the *nfya7* mutant and WT plants still maintained several opened stomata (**Figure 5J**). Overall, these results indicated that *PtNF-YA9* overexpression in *Arabidopsis* confers drought resistance via an increased ABA sensitive to induced stomatal closure.

### Expression Analysis of Stress-Responsive Genes Regulated by the *PtNF-YA9* Transcription Factor

**FIGURE 6:**
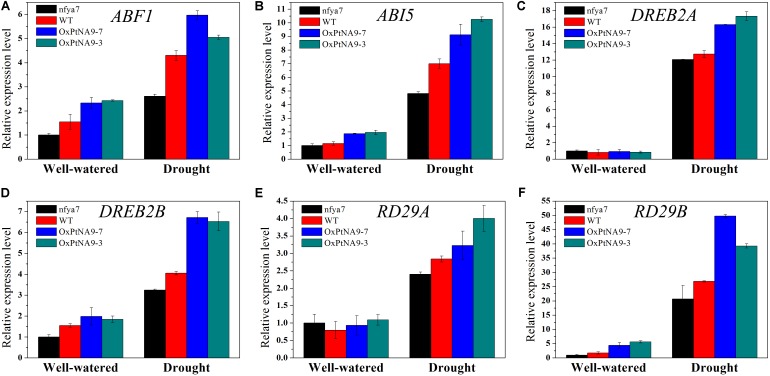
Transcript level analysis of drought-related genes in the leaves of *nfya7* mutant, WT, and three different OxPtNA9 lines under well-watered and drought conditions by RT-qPCR. Under well-watered conditions, the seedlings and leaves were sampled on day 21 after being transferred to soil. Under drought conditions, the seedlings and leaves were sampled after 7 days of water withholding condition. The drought-related genes including *ABF1* **(A)**, *ABI5* **(B)**, *DREB2A* **(C)**, *DREB2B* **(D)**, *RD29A* **(E)**, and *RD29B* **(F)**.

To detect the enhanced drought resistance by altered gene expression level of *PtNF-YA9*, the transcript abundance of some stress-related genes, including ABA-activated signaling pathway genes *ABF1* and *ABI5* (**Figures 6A,B**), dehydration-responsive element binding protein genes *DREB2A* and *DREB2B* (**Figures 6C,D**), and desiccation-responsive genes *RD29A* and *RD29B* (**Figures 6E,F**), in the leaves of *nfya7* mutant, WT, OxPtNA9-7, and OxPtNA9-3 under well-watered and drought conditions were analyzed by RT-qPCR. The results showed that the stress-related genes were differentially expressed in the OxPtNA9 lines compared with the WT and *nfya7* mutant lines. Under well-watered condition, the expression levels of *DREB2A* and *RD29A* in OxPtNA9 lines were similar to WT, and the expression levels of *ABF1*, *ABI5*, *DREB2B*, and *RD29B* were highly expressed. Under water deficit condition for 7 days, these stress-responsive genes were strongly induced. Moreover, the expression levels of these genes were induced higher in OxPtNA9 lines than in WT and *nfya7* plants.

### *PtNF-YA9* Confers Salt Tolerance in Seedling Stage

To detect whether *PtNF-YA9* is also resistant to salt stress, 7-day-old seedlings of *nfya7* mutant, WT, OxPtNA9/nfya7, and OxPtNA9 lines were transferred to 100 mM NaCl of 1/2 MS agar medium. The results indicated a significant difference in the length of the primary root, and OxPtNA9 lines showed much longer primary root than *nfya7*, WT, and OxPtNA9/nfya7 plants (**Supplementary Figures S6A,B**). Furthermore, to examine the different effects of *nfya7*, WT, OxPtNA9/nfya7, and OxPtNA9 lines on long-term salt treatment, we subjected 2-week-old *nfya7*, WT, OxPtNA9/nfya7, and OxPtNA9 plants to salt stress by pouring saltwater every 5 days until significant difference phenotype was achieved. With the salt stress lasting, *nfya7* mutant, WT, and OxPtNA9/nfya7 plants displayed much more serious phenotypes than OxPtNA9 lines. As plants withered, the leaves turned yellow and a portion of them became white. The *nfya7* mutant, WT, and OxPtNA9/nfya7 plants stopped bolting, and almost all lodged, whereas OxPtNA9 lines showed slow growth with erect bolting (**Supplementary Figure S6C**). Taking together, salt treatment studies showed that *PtNF-YA9* confers high-salt tolerance after seedling-established stages.

### *PtNF-YA9* Involved in Plant Growth and Development at Different Stages

**FIGURE 7:**
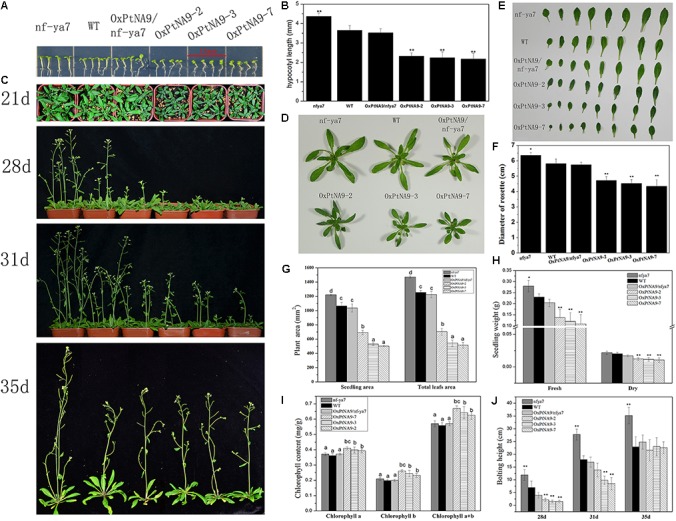
Growth difference of OxPtNA9 lines at different stages. **(A)** Morphology difference of hypocotyl of 10-day-old seedlings. **(B)** Hypocotyl length statistics. **(C)** Phenotypes of differences in *Arabidopsis* growth process. **(D)** Morphology of rosettes of 3-week-old seedlings. **(E)** Morphology of detached leaves of 3-week-old seedlings. **(F)** Diameter of rosettes of 3-week-old seedlings. **(G)** Plant area of 3-week-old seedlings. **(H)** Fresh and dry weights of 3-week-old seedlings. **(I)** Chlorophyll a and b and total chlorophyll contents of 3-week-old seedlings. **(J)** Height of inflorescence at different days.

**FIGURE 8:**
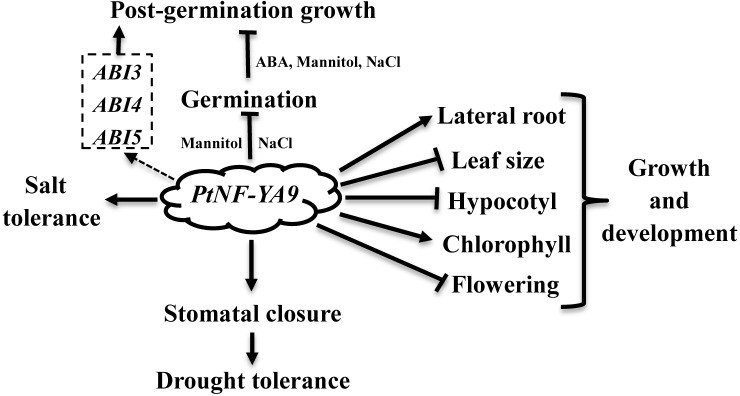
*PtNF-YA9* plays pleiotropic functions in *Arabidopsis*. Dashed boxes are genes.

*PtNF-YA9* altered the lateral root growth (**Supplementary Figure S4**) and exhibited a dwarf phenotype (**Figures 4**, **5**) both at the seedling from post-germination growth and adult stages (**Figures 8**). A very obvious phenotype is the shortened hypocotyl in the seedling stage of *PtNF-YA9* overexpressing lines (**Figure 7A**). The *nfya7* mutant lines had the longest length of hypocotyl (4.37 ± 0.17 mm), WT showed the middle phenotype (3.65 ± 0.25 mm), whereas OxPtNA9 lines had the significantly shortest length of hypocotyl (1.87–2.60 mm) (**Figure 7B**).

Another dwarf phenotype of OxPtNA9 lines was reflected in the diameter and the area of OxPtNA9 rosettes, which were markedly reduced (**Figures 7C–G**). Three lines of OxPtNA9 seedling area were 693.08 ± 30.97, 529.65 ± 20.02, and 502.71 ± 11.04 mm^2^, showing almost half the area of *nfya7* mutant seedlings, 1220.42 ± 10.22 mm^2^. WT and OxPtNA9/nfya7 complementary lines showed the middle phenotypes of approximately 1063.05 ± 52.42 and 1036.44 ± 57.31 mm^2^, respectively. Calculating the sum of all detached leaf areas also showed similar results (**Figure 7G**). The dwarf phenotype also resulted in biomass reduction. Both the fresh and dry weights of *nfya7* mutant lines were heavier than WT, whereas the weight of OxPtNA9 lines was significantly low (**Figure 7H**).

In addition, the leaves of OxPtNA9 lines exhibited darker green color than WT. This phenomenon correlated with a higher chlorophyll content of OxPtNA9 lines compared with WT (**Figure 7I**). It indicated significantly higher chlorophyll a (Ca: 0.39–0.41 mg/g) and chlorophyll b (Cb: 0.23–0.26 mg/g) in the OxPtNA9 lines than in WT (Ca: 0.36 mg/g, Cb: 0.19 mg/g) and *nfya7* mutant plants (Ca: 0.37 mg/g, Cb: 0.20 mg/g), resulting in a significantly increased total chlorophyll content of OxPtNA9 lines.

To assay the bolted and flowering time, the *nfya7* mutant, WT, and OxPtNA9 lines were planted and grown under similar conditions. The result showed that the WT bolted at 24–25 days, and *nfya7* mutant plants bolted early at 22–23 days. Meanwhile, the OxPtNA9 lines bolted at 26–27 days after sowing the seeds (**Figure 7C**). Additionally, the *nfya7* mutant plants showed higher stem elongation at the early shooting stage compared with WT, and OxPtNA9 lines showed the lowest stem elongation. At the 31st day, the inflorescence length of OxPtNA9 lines varied from 6.2 cm to 16.8 cm, WT varied from 16.2 cm to 20.2 cm, and the *nfya7* mutant varied from 24.3 cm to 30.2 cm. However, at the 35th day, the inflorescence length of OxPtNA9 lines was 17.0–27.0 cm and exhibited no significant difference compared with WT, 18.2–27.9 cm. Meanwhile, the *nfya7* mutant lines also had high inflorescence of 30.6–39.4 cm (**Figure 7J**). These results indicated that overexpression of *PtNF-YA9* lines exhibits slow stem elongation at the early shooting stage and delayed flowering.

## Discussion

*Populus trichocarpa NF-YA* is an unknown function member of the *NF-Y* family. In this study, the roles of *PtNF-YA9* at different stages were reported comprehensively. At the germination stage, the *PtNF-YA9* overexpressing lines are hypersensitive to mannitol, salt, and ABA treatments and exhibited growth arrest during the post-germination stage (**Figure 4**). At seedling stages, OxPtNA9 lines showed dwarf phenotype of short hypocotyl, small leaf, less biomass, and delayed flowering but promoted the lateral root growth and increased chlorophyll content. These different observations and multiple functions demonstrated that *PtNF-YA9* participates in multiple signaling pathways at different growth stages (**Figure 8**).

### *PtNF-YA9* Negatively Regulates Seed Germination Involved in ABA Signaling Pathway

At the seed germination and post-germination growth stages, OxPtNA9 lines showed hypersensitive phenotype to salt, ABA, and mannitol stresses. The germination rate of OxPtNA9 lines was decreased and delayed, and dramatic arrest development of the post-germination growth before the cotyledons turned green and developed into seedlings under the treatment of ABA, mannitol, and NaCl treatment (**Figure 4**). Lopez-Molina et al. found that post-germination growth arrest is one of the adverse strategies to protect the germinated seeds from water deficit, and the application of ABA can also result in growth arrest after germination (Lopez-Molina et al., 2001). Both mannitol and NaCl treatments could cause water deficit and induce ABA production. Thus, *PtNF-YA9* played a positive role to protect the germinated seeds from water deficit under abiotic stress by arresting growth after germination. Genes of *cpr5* (Gao et al., 2011), *wrky2* (Jiang and Yu, 2009), and *hyl1* (Lu et al., 2002) were also involved in ABA signaling pathways and participated in the regulation of post-germination growth arrest. In *Arabidopsis*, *AtNF-YA1* also regulates post-germination growth arrest and is involved in ABA signaling pathways under salt stress (Li et al., 2013).

Abscisic acid, an important stress phytohormone, not only acted as a pivotal signaling molecule in stomatal movement and abiotic stress but was also involved in seed germination and post-germination growth. In this study, to reveal whether the post-germination growth arrest of *PtNF-YA9* was also involved in ABA signaling, the ABA treatment of OxPtNA9 seeds was further studied, and the result showed that ABA acted positively in post-germination growth arrest under exogenous ABA treatment (**Figure 4C**). *ABI3* and *ABI5*, as important genes in the ABA signaling pathway, play an important regulatory role in the growth arrest during seed germination and post-germination growth (Lopez-Molina et al., 2001). *ABI5* played a positive role in keeping germinated seeds in a quiescent state to protect them from drought stress (Lopez-Molina et al., 2002). *ABI4* directly promotes *ABI5* transcription (Bossi et al., 2010), positively regulates primary seed dormancy, while negatively regulating cotyledon greening, by mediating the biogenesis of ABA and GA (Shu et al., 2013). Furthermore, to acquire further insight into the mechanism of *PtNF-YA9* during germination stage, the transcript abundances of the *ABI3*, *ABI4*, and *ABI5* in OxPtNA9 lines under abiotic stresses were analyzed. Interestingly, the transcript abundances of *ABI3*, *ABI4*, and *ABI5* were significantly increased in OxPtNA9 lines under abiotic stresses (**Figure 4E**). The high expression of *ABI3*, *ABI4*, and *ABI5* in OxPtNA9 lines under abiotic stress might stimulate post-germination growth arrest. In conclusion, our study revealed that during the post-germination growth arrest, *PtNF-YA9* might participate in the regulation of the ABA signaling pathway via the regulation of *ABIs*.

### *PtNF-YA9* Confers High Drought Tolerance Through Affecting Stomatal Aperture

Plants have complicated signaling regulatory mechanisms to acclimatize in adverse environments. Many transcription factors had been reported to bind specifically the motif in the promoters of the stress-responsive genes and are involved in drought tolerance. Some *NF-YA* genes had been identified as regulators of drought stress in different plants. *AtNF-YA5* (Li et al., 2008), *GmNF-YA3* (Ni et al., 2013), and *OsHAP2E* (Alam et al., 2015) were demonstrated to enhance drought tolerance in *Arabidopsis*, soybeans, and rice, respectively. In our study, *PtNF-YA9* overexpression in *Arabidopsis* exhibited typical drought-resistant phenotypes, such as dwarf phenotype, small seedling areas, increased chlorophyll content, high photosynthetic rate, and improved WUE under drought condition. Substantial evidence demonstrated that growth reduction is a part of an acclimatization strategy in plants to adapt to adversity. *CBF1* (Achard et al., 2008), *AtNF-YA5* (Li et al., 2008), *AtNF-YA2*, *AtNF-YA7*, and *AtNF-YA10* genes (Leyva-González et al., 2012) all exhibited dwarfism phenotypes and confers drought stress. Based on the phylogenetic analysis, *PtNF-YA9* clustered with *AtNF-YA7*, and they shared high similarity. Consistent with this observation, *PtNF-YA9* overexpression in *Arabidopsis* also caused dwarf phenotype and enhanced drought tolerance at the vegetative stage (**Figure 5A**).

Osmotic stress induces a large number of stress-response genes and is involved in an ABA-dependent or ABA-independent manner (Yoshida et al., 2014). In an ABA-dependent manner, endogenous ABA was induced to modulate stomatal aperture under drought stress, and the expression levels of many drought-response genes were also altered (Zhu, 2002; Seki et al., 2007). RT-qPCR analysis showed that *PtNF-YA9* expression was downregulated under ABA treatment (**Figure 3**). Moreover, seed germination assay provided further evidence to support that *PtNF-YA9* is involved in ABA regulation in stress tolerance (**Figure 4C**). Thus, whether *PtNF-YA9*-enhanced drought tolerance in *Arabidopsis* was regulated by ABA to induce stomatal closure remains to be elucidated. To clarify this issue, first, water loss assay was performed and revealed that OxPtNA9 lines enhance water retention (**Figure 5D**). Second, OxPtNA9 lines promoted stomatal closure in *Arabidopsis* under ABA treatment (**Figure 5J**). Third, the expression of ABA-related genes *ABI5* and *ABF* was significantly changed in the OxPtNA9 lines compared with WT both at normal and drought conditions (**Figure 6**). In conclusion, our results indicated that *PtNF-YA9* transgenic plants confer drought tolerance by reducing water loss and promoting stomatal closure via the ABA signaling pathway.

### *PtNF-YA9* Has a Negative Effect on Plant Growth

In *Arabidopsis*, most *NF-YA* overexpressing lines exhibited dwarf phenotype with smaller rosette diameters than WT plants except *NF-YA4* and *NF-YA6* (Siriwardana et al., 2014). In this study, we found that OxPtNA9 lines also had dwarf phenotype (**Figure 7**), short hypocotyl (**Figure 7A**), small leaf area and biomass (**Figures 7C–G**), and delayed flowering (**Figure 7C**). Plant growth predominantly depends on cellular elongation, and *EXPs* and *XTHs* play an important role in cell wall loosening with realignment of cell wall components (Cosgrove, 2005). In addition, *XTH* and *EXP* genes were repressed in several PXVE:NF-YA lines (Leyva-González et al., 2012). Similar to PXVE:NF-YA lines in *Arabidopsis* (Leyva-González et al., 2012), *PtNF-YA9* or the *PtNF-YA9* heterotrimeric complex might participate in *XTHs* and *EXPs* to regulate cell elongation.

*AtNF-YA2* and *AtNF-YA10* promoting leaf development and cell expansion were regulated by the auxin signaling pathway. Auxin homeostasis, polar transport, and signaling affect the entire progress of leaf development (Scarpella et al., 2010). Mutations of *ARF19* and *NPH4*/*ARF7* interact with each other and cause a reduced leaf cell expansion (Wilmoth et al., 2005). Furthermore, during leaf–primordium development, auxin production shifts and plays an important role in leaf morphogenesis and vascular differentiation (Mattsson et al., 1999; Aloni et al., 2003). Previous findings suggested that *MP*, *IAA12/BDL*, and *AXR6* genes participate in auxin signal transduction during vascular development (Hamann et al., 2002). In our studies, *PtNF-YA9* altered the leaf size, and GUS staining of ProNF-YA9::GUS showed especially high expression level patterns in leaf vasculature. Thus, *PtNF-YA9* might mediate the expression of auxin-related genes to alter the leaf size. However, several additional experiments are required to directly demonstrate the interaction of *PtNF-YA9* with auxin-related genes.

In conclusion, *PtNF-YA9* plays pleiotropic functions, such as seed germination, post-germination growth, abiotic stress, plant growth, and development (**Figure 8**). The CCAAT-box element, which presents approximately 30% of eukaryotic promoter sequence, can be recognized by *NF-Y* activating transcription (Bucher, 1990). *NF-Ys* are encoded by three multigene subfamilies, which contain 10 *NF-YAs*, 13 *NF-YBs*, and 13 *NF-YCs* genes in *Arabidopsis* (Siefers et al., 2009). With *PtNF-YA9* alone or heterotrimeric complex formed in *Arabidopsis*, possibilities of at least 1 × 13 × 13 combinations would exist. Thus, the pleiotropic functions of *PtNF-YA9* can be explained by the various combinations of *PtNF-YA9* with other *NF-YB/NF-YC* factors at different stages in *Arabidopsis*. In a word, it is meaningful to gain deep insight into the molecular mechanism of *PtNF-YA9* at different stages, and further investigation on the interactions of protein–DNA or protein–protein is also needed to establish *PtNF-YA9* regulation network at each individual function.

## Conclusion

We have demonstrated that *PtNF-YA9* plays an important role in the regulation of drought stress in *Arabidopsis* via the ABA-mediated signaling pathway. In our gain-of-function genetic studies, *PtNF-YA9* overexpressing lines were hypersensitive to simulated drought, ABA, and salt stresses during the early post-germination growth stages and showed a severe post-germination growth arrest involved in ABA signaling pathway by elevating the expression levels of *ABI3* and *ABI5*. Meanwhile, *PtNF-YA9* overexpressing lines displayed high instantaneous leaf WUE and decreased in water loss via ABA-induced stomatal closure to exhibit enhanced drought tolerance at seedling stages. In addition, OxPtNA9 lines also confer strong tolerance to salt stress. Furthermore, *PtNF-YA9* was also involved in the regulation of plant growth and development in exhibiting dwarf phenotype, as reduced hypocotyl, leaf area, and biomass and delayed flowering, while promoting lateral root growth and increasing chlorophyll content. Our findings propose a model in which *PtNF-YA9* plays an important role in reducing plant growth and provide a valuable and complex insight into the plant adaption to abiotic stress.

## Author Contributions

CL, WY, and XX conceived the research and designed the experiments. CL performed the experiments with help from KY, YZ, and QL. CL analyzed the experimental results with the assistance of SM. All authors discussed the results. CL and XX wrote the manuscript. And all the authors approved the final manuscript.

## Conflict of Interest Statement

The authors declare that the research was conducted in the absence of any commercial or financial relationships that could be construed as a potential conflict of interest.

## Footnotes

1 http://popgenie.org/gene?id=Potri.011G101000

2 http://www.ebi.ac.uk/interpro/

3 http://web.expasy.org/protparam/

4 http://popgenie.org/

## Supplementary Material

FIGURE S1 Phylogenetic tree analysis of the NF-YA subfamily proteins in *P. trichocarpa* and *Arabidopsis*. The amino acid sequences were aligned using MAFFT, and the phylogenetic tree was constructed using MEGA 6.0 software by neighbor-joining method with bootstrap analysis of 1000 replicates.

FIGURE S2 Promoter sequence and *cis*-element analysis of *PtNF-YA9*. Arrow lines are *cis*-elements, and the direction represents sense and antisense strand. ATG is the translation initiation site, and the predicted core promoter region is marked by the black box.

FIGURE S3 Molecular analysis of *PtNF-YA9* transgenic *Arabidopsis* plants via RT-PCR and RT-qPCR. **(A)** Simple diagram of 35S:PtNF-YA9 vector. **(B,C)** mRNA level identification via RT-PCR **(B)** and RT-qPCR **(C)** of *PtNF-YA9* overexpression lines in *Arabidopsis*.

FIGURE S4 Overexpression of *PtNF-YA9* grown under 1/2 MS agar medium at seedling stage in *Arabidopsis*. **(A)** Morphological differences of root phenotypes in 1/2 MS agar medium plates. **(B)** Primary root length calculated at different lines. **(C)** Lateral root numbers calculated at different lines.

FIGURE S5 Overexpression of *PtNF-YA9* confers drought tolerance at seedling stage in *Arabidopsis*. **(A)** Morphological differences in 1/2 MS agar medium plates with 200 mM mannitol. **(B)** Primary root length of different plants calculated under treatment of 200 mM mannitol.

FIGURE S6 *PtNF-YA9* overexpression confers salt tolerance in *Arabidopsis*. **(A)** Morphological differences in 1/2 MS agar medium plates with 100 mM NaCl. **(B)** Primary root length of different plants calculated under treatment of 100 mM NaCl. **(C)** Morphological differences in long-term salt treatment. The seedlings were grown in soil for 2 weeks under well-watered conditions and thereafter subjected to salt stress by pouring 100 mM NaCl–water every 5 days until 4 weeks.

TABLE S1 Primers used in this research.

## References

[B1] Achard P. Gong F. Cheminant S. Alioua M. Hedden P. Genschik P. (2008). The cold-inducible CBF1 factor-dependent signaling pathway modulates the accumulation of the growth-repressing DELLA proteins via its effect on gibberellin metabolism. *Plant Cell* 20 2117–2129. 10.1105/tpc.108.058941 18757556 PMC2553604

[B2] Ahn H. Jung I. Shin S. J. Park J. Rhee S. Kim J. K. (2017). Transcriptional network analysis reveals drought resistance mechanisms of AP2/ERF transgenic rice. *Front. Plant Sci.* 8:1044. 10.3389/fpls.2017.01044 28663756 PMC5471331

[B3] Alam M. M. Tanaka T. Nakamura H. Ichikawa H. Kobayashi K. Yaeno T. (2015). Overexpression of a rice heme activator protein gene (OsHAP2E) confers resistance to pathogens, salinity and drought, and increases photosynthesis and tiller number. *Plant Biotechnol. J.* 13 85–96. 10.1111/pbi.12239 25168932

[B4] Aloni R. Schwalm K. Langhans M. Ullrich C. I. (2003). Gradual shifts in sites of free-auxin production during leaf-primordium development and their role in vascular differentiation and leaf morphogenesis in Arabidopsis. *Planta* 216 841–853. 12624772 10.1007/s00425-002-0937-8

[B5] Bossi F. Cordoba E. Dupré P. Mendoza M. S. Román C. S. León P. (2010). The Arabidopsis ABA-INSENSITIVE (ABI) 4 factor acts as a central transcription activator of the expression of its own gene, and for the induction of *ABI5* and *SBE2.2* genes during sugar signaling. *Plant J.* 59 359–374. 10.1111/j.1365-313X.2009.03877.x 19392689

[B6] Bucher P. (1990). Weight matrix descriptions of four eukaryotic RNA polymerase II promoter elements derived from 502 unrelated promoter sequences. *J. Mol. Biol.* 212 563–578. 10.1016/0022-2836(90)90223-9 2329577

[B7] Burke E. J. Brown S. J. Christidis N. (2006). Modeling the recent evolution of global drought and projections for the twenty-first century with the hadley centre climate model. *J. Hydrometeorol.* 7 1113–1125. 10.1175/Jhm544.1

[B8] Cao S. Kumimoto R. W. Gnesutta N. Calogero A. M. Mantovani R. Holt B. F. III (2014). A distal *CCAAT*/NUCLEAR FACTOR Y complex promotes chromatin looping at the *FLOWERING LOCUS T* promoter and regulates the timing of flowering in *Arabidopsis*. *Plant Cell* 26 1009–1017. 10.1105/tpc.113.120352 24610724 PMC4001365

[B9] Clough S. J. Bent A. F. (1998). Floral dip: a simplified method for *Agrobacterium*-mediated transformation of *Arabidopsis thaliana*. *Plant J.* 16 735–743. 10.1046/j.1365-313x.1998.00343.x 10069079

[B10] Cosgrove D. J. (2005). Growth of the plant cell wall. *Nat. Rev. Mol. Cell Biol.* 6 850–861. 10.1038/nrm1746 16261190

[B11] Ding Q. Zeng J. He X. Q. (2016). MiR169 and its target PagHAP2-6 regulated by ABA are involved in poplar cambium dormancy. *J. Plant Physiol.* 198 1–9. 10.1016/j.jplph.2016.03.017 27111502

[B12] Dong Y. Wang C. Han X. Tang S. Liu S. Xia X. (2014). A novel bHLH transcription factor *PebHLH35* from *Populus euphratica* confers drought tolerance through regulating stomatal development, photosynthesis and growth in *Arabidopsis*. *Biochem. Biophys. Res. Commun.* 450 453–458. 10.1016/j.bbrc.2014.05.139 24909687

[B13] Forsburg S. L. Guarente L. (1989). Identification and characterization of HAP4: a third component of the CCAAT-bound HAP2/HAP3 heteromer. *Genes Dev.* 3 1166–1178. 10.1101/gad.3.8.1166 2676721

[B14] Frontini M. Imbriano C. Manni I. Mantovani R. (2004). Cell cycle regulation of NF-YC nuclear localization. *Cell Cycle* 3 217–222. 10.4161/cc.3.2.654 14712092

[B15] Gao G. Zhang S. Wang C. Yang X. Wang Y. Su X. (2011). Arabidopsis CPR5 independently regulates seed germination and postgermination arrest of development through LOX pathway and ABA signaling. *PLoS One* 6:e19406. 10.1371/journal.pone.0019406 21556325 PMC3083440

[B16] Hamann T. Benkova E. Baurle I. Kientz M. Jurgens G. (2002). The Arabidopsis BODENLOS gene encodes an auxin response protein inhibiting MONOPTEROS-mediated embryo patterning. *Genes Dev.* 16 1610–1615. 10.1101/gad.229402 12101120 PMC186366

[B17] Han X. Tang S. An Y. Zheng D.-C. Xia X.-L. Yin W.-L. (2013). Overexpression of the poplar *NF-YB7* transcription factor confers drought tolerance and improves water-use efficiency in *Arabidopsis*. *J. Exp. Bot.* 64 4589–4601. 10.1093/jxb/ert262 24006421 PMC3808328

[B18] Jiang W. Yu D. (2009). Arabidopsis WRKY2 transcription factor mediates seed germination and postgermination arrest of development by abscisic acid. *BMC Plant Biol.* 9:96. 10.1186/1471-2229-9-96 19622176 PMC2719644

[B19] Jiang Y. Duan Y. Yin J. Ye S. Zhu J. Zhang F. (2014). Genome-wide identification and characterization of the *Populus* WRKY transcription factor family and analysis of their expression in response to biotic and abiotic stresses. *J. Exp. Bot.* 65 6629–6644. 10.1093/jxb/eru381 25249073 PMC4246191

[B20] Jin J. Tian F. Yang D. C. Meng Y. Q. Kong L. Luo J. (2017). PlantTFDB 4.0: toward a central hub for transcription factors and regulatory interactions in plants. *Nucleic Acids Res.* 45 D1040–D1045. 10.1093/nar/gkw982 27924042 PMC5210657

[B21] Lee H. Fischer R. L. Goldberg R. B. Harada J. J. (2003). *Arabidopsis* LEAFY COTYLEDON1 represents a functionally specialized subunit of the CCAAT binding transcription factor. *Proc. Natl. Acad. Sci. U.S.A.* 100 2152–2156. 10.1073/pnas.0437909100 12578989 PMC149974

[B22] Lescot M. Dehais P. Thijs G. Marchal K. Moreau Y. Van de Peer Y. (2002). PlantCARE, a database of plant cis-acting regulatory elements and a portal to tools for in silico analysis of promoter sequences. *Nucleic Acids Res.* 30 325–327. 10.1093/nar/30.1.325 11752327 PMC99092

[B23] Leyva-González M. A. Ibarra-Laclette E. Cruz-Ramírez A. Herrera-Estrella L. (2012). Functional and transcriptome analysis reveals an acclimatization strategy for abiotic stress tolerance mediated by *Arabidopsis* NF-YA family members. *PLoS One* 7:e48138. 10.1371/journal.pone.0048138 23118940 PMC3485258

[B24] Li L. Zheng W. Zhu Y. Ye H. Tang B. Arendsee Z. W. (2015). QQS orphan gene regulates carbon and nitrogen partitioning across species via NF-YC interactions. *Proc. Natl. Acad. Sci. U.S.A.* 112 14734–14739. 10.1073/pnas.1514670112 26554020 PMC4664325

[B25] Li W. X. Oono Y. Zhu J. He X. J. Wu J. M. Iida K. (2008). The *Arabidopsis* NFYA5 transcription factor is regulated transcriptionally and posttranscriptionally to promote drought resistance. *Plant Cell* 20 2238–2251. 10.1105/tpc.108.059444 18682547 PMC2553615

[B26] Li X. (2011). Infiltration of *Nicotiana benthamiana* protocol for transient expression via *Agrobacterium*. *Bio Protoc.* 1:e95 10.21769/BioProtoc.95

[B27] Li Y.-J. Fang Y. Fu Y.-R. Huang J.-G. Wu C.-A. Zheng C.-C. (2013). NFYA1 is involved in regulation of postgermination growth arrest under salt stress in *Arabidopsis*. *PLoS One* 8:e61289. 10.1371/journal.pone.0061289 23637805 PMC3634844

[B28] Lopez-Molina L. Mongrand S. Chua N. H. (2001). A postgermination developmental arrest checkpoint is mediated by abscisic acid and requires the ABI5 transcription factor in *Arabidopsis*. *Proc. Natl. Acad. Sci. U.S.A.* 98 4782–4787. 10.1073/pnas.081594298 11287670 PMC31911

[B29] Lopez-Molina L. Mongrand S. McLachlin D. T. Chait B. T. Chua N. H. (2002). ABI5 acts downstream of ABI3 to execute an ABA-dependent growth arrest during germination. *Plant J.* 32 317–328. 10.1046/j.1365-313X.2002.01430.x 12410810

[B30] Lu C. Han M. H. Guevara-Garcia A. Fedoroff N. V. (2002). Mitogen-activated protein kinase signaling in postgermination arrest of development by abscisic acid. *Proc. Natl. Acad. Sci. U.S.A.* 99 15812–15817. 10.1073/pnas.242607499 12434021 PMC137798

[B31] Lu X. Zhang X. Duan H. Lian C. Yin W. Xia X. (2017). Three stress-responsive NAC transcription factors from *Populus euphratica* differentially regulate salt and drought tolerance in transgenic plants. *Physiol. Plant.* 162 73–97. 10.1111/ppl.12613 28776695

[B32] Ma X. Zhu X. Li C. Song Y. Zhang W. Xia G. (2015). Overexpression of wheat NF-YA10 gene regulates the salinity stress response in *Arabidopsis thaliana*. *Plant Physiol. Biochem.* 86 34–43. 10.1016/j.plaphy.2014.11.011 25461698

[B33] Mattsson J. Sung Z. R. Berleth T. (1999). Responses of plant vascular systems to auxin transport inhibition. *Development* 126 2979–2991. 10357941 10.1242/dev.126.13.2979

[B34] Ni Z. Y. Hu Z. Jiang Q. Y. Zhang H. (2013). GmNFYA3, a target gene of miR169, is a positive regulator of plant tolerance to drought stress. *Plant Mol. Biol.* 82 113–129. 10.1007/s11103-013-0040-5 23483290

[B35] Pant B. D. Musialak-Lange M. Nuc P. May P. Buhtz A. Kehr J. (2009). Identification of nutrient-responsive Arabidopsis and rapeseed microRNAs by comprehensive real-time polymerase chain reaction profiling and small RNA sequencing. *Plant Physiol.* 150 1541–1555. 10.1104/pp.109.139139 19465578 PMC2705054

[B36] Potkar R. Recla J. Busov V. (2013). ptr-MIR169 is a posttranscriptional repressor of PtrHAP2 during vegetative bud dormancy period of aspen (*Populus tremuloides*) trees. *Biochem. Biophys. Res. Commun.* 431 512–518. 10.1016/j.bbrc.2013.01.027 23321309

[B37] Sade D. Sade N. Shriki O. Lerner S. Gebremedhin A. Karavani A. (2014). Water balance, hormone homeostasis, and sugar signaling are all involved in tomato resistance to tomato yellow leaf curl virus. *Plant Physiol.* 165 1684–1697. 10.1104/pp.114.243402 24989233 PMC4119048

[B38] Scarpella E. Barkoulas M. Tsiantis M. (2010). Control of leaf and vein development by auxin. *Cold Spring Harb. Perspect. Biol.* 2:a001511. 10.1101/cshperspect.a001511 20182604 PMC2827905

[B39] Seki M. Umezawa T. Urano K. Shinozaki K. (2007). Regulatory metabolic networks in drought stress responses. *Curr. Opin. Plant Biol.* 10 296–302. 10.1016/j.pbi.2007.04.014 17468040

[B40] Shu K. Zhang H. Wang S. Chen M. Wu Y. Tang S. (2013). ABI4 regulates primary seed dormancy by regulating the biogenesis of abscisic acid and gibberellins in Arabidopsis. *PLoS Genet.* 9:e1003577. 10.1371/journal.pgen.1003577 23818868 PMC3688486

[B41] Siefers N. Dang K. K. Kumimoto R. W. Bynum W. E. IV Tayrose G. Holt B. F. III (2009). Tissue-specific expression patterns of Arabidopsis NF-Y transcription factors suggest potential for extensive combinatorial complexity. *Plant Physiol.* 149 625–641. 10.1104/pp.108.130591 19019982 PMC2633833

[B42] Siriwardana C. L. Kumimoto R. W. Jones D. S. Holt B. F. III (2014). Gene family analysis of the *Arabidopsis NF-YA* transcription factors reveals opposing abscisic acid responses during seed germination. *Plant Mol. Biol. Rep.* 32 971–986. 10.1007/s11105-014-0704-6 25190903 PMC4149875

[B43] Sorin C. Declerck M. Christ A. Blein T. Ma L. Lelandais-Briere C. (2014). A miR169 isoform regulates specific NF-YA targets and root architecture in Arabidopsis. *New Phytol.* 202 1197–1211. 10.1111/nph.12735 24533947

[B44] Steibel J. Poletto R. Rosa G. (2005). Statistical analysis of relative quantification of gene expression using real time RT-PCR data. *J. Anim. Sci.* 83 104–104.

[B45] Steidl S. Tuncher A. Goda H. Guder C. Papadopoulou N. Kobayashi T. (2004). A single subunit of a heterotrimeric CCAAT-binding complex carries a nuclear localization signal: piggy back transport of the pre-assembled complex to the nucleus. *J. Mol. Biol.* 342 515–524. 10.1016/j.jmb.2004.07.011 15327951

[B46] Tuskan G. A. Difazio S. Jansson S. Bohlmann J. Grigoriev I. Hellsten U. (2006). The genome of black cottonwood, *Populus trichocarpa* (Torr. & Gray). *Science* 313 1596–1604. 10.1126/science.1128691 16973872

[B47] Valliyodan B. Nguyen H. T. (2006). Understanding regulatory networks and engineering for enhanced drought tolerance in plants. *Curr. Opin. Plant Biol.* 9 189–195. 10.1016/j.pbi.2006.01.019 16483835

[B48] Wei Q. Luo Q. Wang R. Zhang F. He Y. Zhang Y. (2017). A wheat R2R3-type MYB transcription factor TaODORANT1 positively regulates drought and salt stress responses in transgenic tobacco plants. *Front. Plant Sci.* 8:1374. 10.3389/fpls.2017.01374 28848578 PMC5550715

[B49] Wilmoth J. C. Wang S. C. Tiwari S. B. Joshi A. D. Hagen G. Guilfoyle T. J. (2005). NPH4/ARF7 and ARF19 promote leaf expansion and auxin-induced lateral root formation. *Plant J.* 43 118–130. 10.1111/j.1365-313X.2005.02432.x 15960621

[B50] Yoshida T. Mogami J. Yamaguchi-Shinozaki K. (2014). ABA-dependent and ABA-independent signaling in response to osmotic stress in plants. *Curr. Opin. Plant Biol.* 21 133–139. 10.1016/j.pbi.2014.07.009 25104049

[B51] Zhu J. K. (2002). Salt and drought stress signal transduction in plants. *Annu. Rev. Plant Biol.* 53 247–273. 10.1146/annurev.arplant.53.091401.143329 12221975 PMC3128348

