# Corrigendum: *Populus trichocarpa PtNF-YA9*, a Multifunctional Transcription Factor, Regulates Seed Germination, Abiotic Stress, Plant Growth and Development in *Arabidopsis*

**DOI:** 10.3389/fpls.2018.01403

**Published:** 2018-09-25

**Authors:** Conglong Lian, Qing Li, Kun Yao, Ying Zhang, Sen Meng, Weilun Yin, Xinli Xia

**Affiliations:** ^1^Beijing Advanced Innovation Center for Tree Breeding by Molecular Design, Beijing Forestry University, Beijing, China; ^2^National Engineering Laboratory for Tree Breeding, Beijing Forestry University, Beijing, China; ^3^College of Biological Sciences and Technology, Beijing Forestry University, Beijing, China; ^4^Key Laboratory of Genetics and Breeding in Forest Trees and Ornamental Plants, Beijing Forestry University, Beijing, China

**Keywords:** *Populus trichocarpa*, *NF-YA*, post-germination growth arrest, drought tolerance, plant development

There is an error in the Funding statement. The correct number for the National Key Program on Transgenic Research is 2018ZX08020002. The authors apologize for this error and state that this does not change the scientific conclusions of the article in any way.

The original article has been updated.

## Conflict of interest statement

The authors declare that the research was conducted in the absence of any commercial or financial relationships that could be construed as a potential conflict of interest.

