# Supplementary figures and images for: Populus trichocarpa PtNF-YA9, A Multifunctional Transcription Factor, Regulates Seed Germination, Abiotic Stress, Plant Growth and Development in Arabidopsis
Source: Front Plant Sci. 2018 Jul 9;9:954. doi: 10.3389/fpls.2018.00954 (PMC6052803; doi:10.3389/fpls.2018.00954)

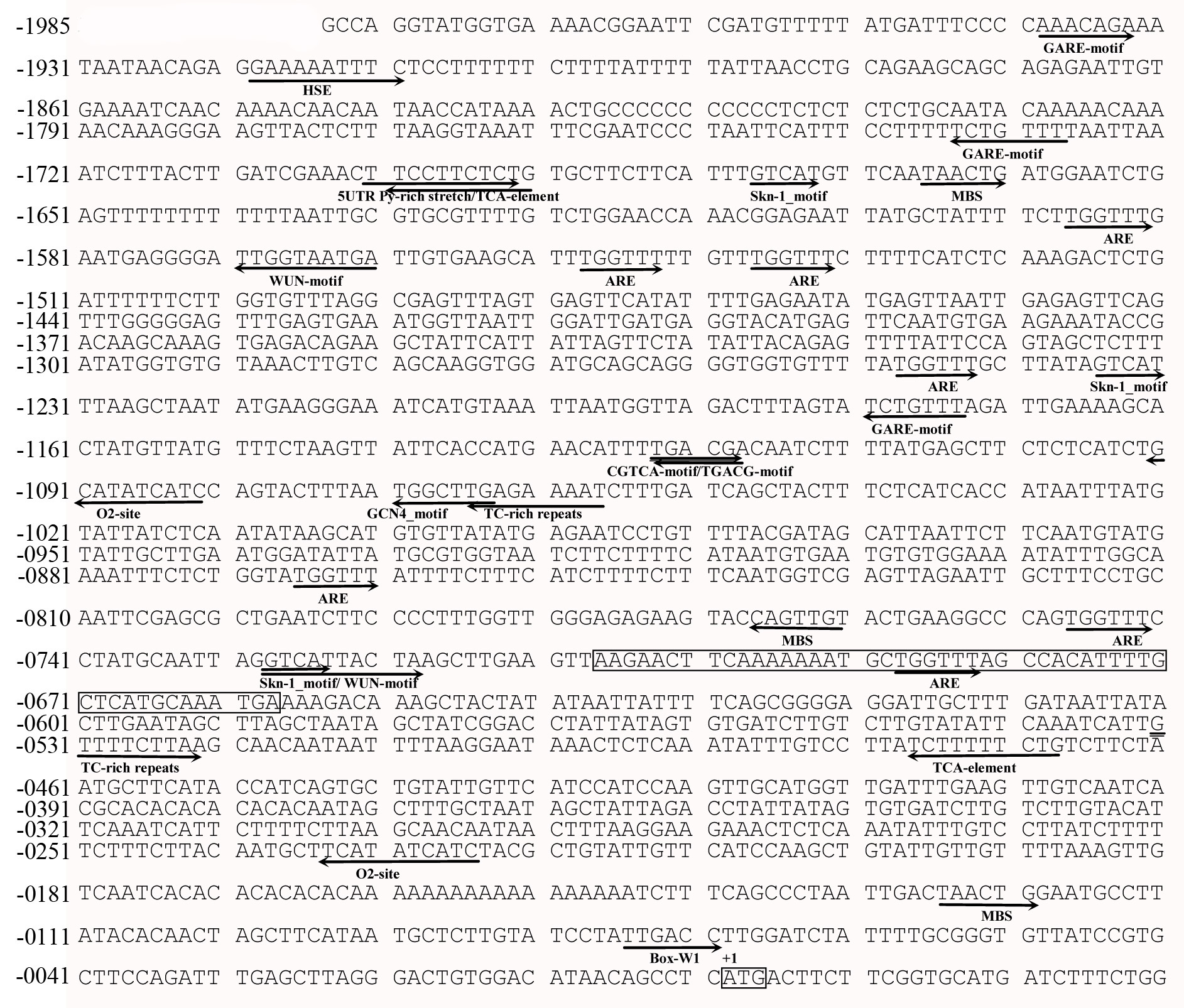

Supplement: FIGURE S2 — Promoter sequence and cis-element analysis of PtNF-YA9. Arrow lines are cis-elements, and the direction represents sense and antisense strand. ATG is the translation initiation site, and the predicted core promoter region is marked by the black box. [file Image_2.JPEG]

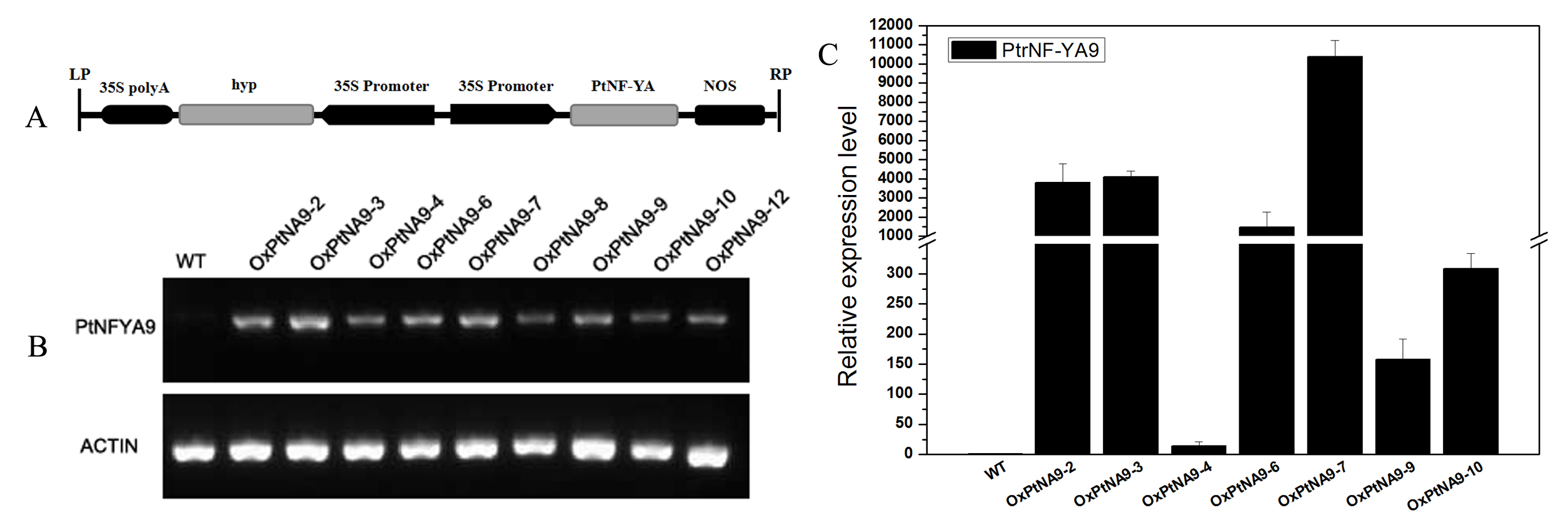

Supplement: FIGURE S3 — Molecular analysis of PtNF-YA9 transgenic Arabidopsis plants via RT-PCR and RT-qPCR. (A) Simple diagram of 35S:PtNF-YA9 vector. (B,C) mRNA level identification via RT-PCR (B) and RT-qPCR (C) of PtNF-YA9 overexpression lines in Arabidopsis. [file Image_3.JPEG]

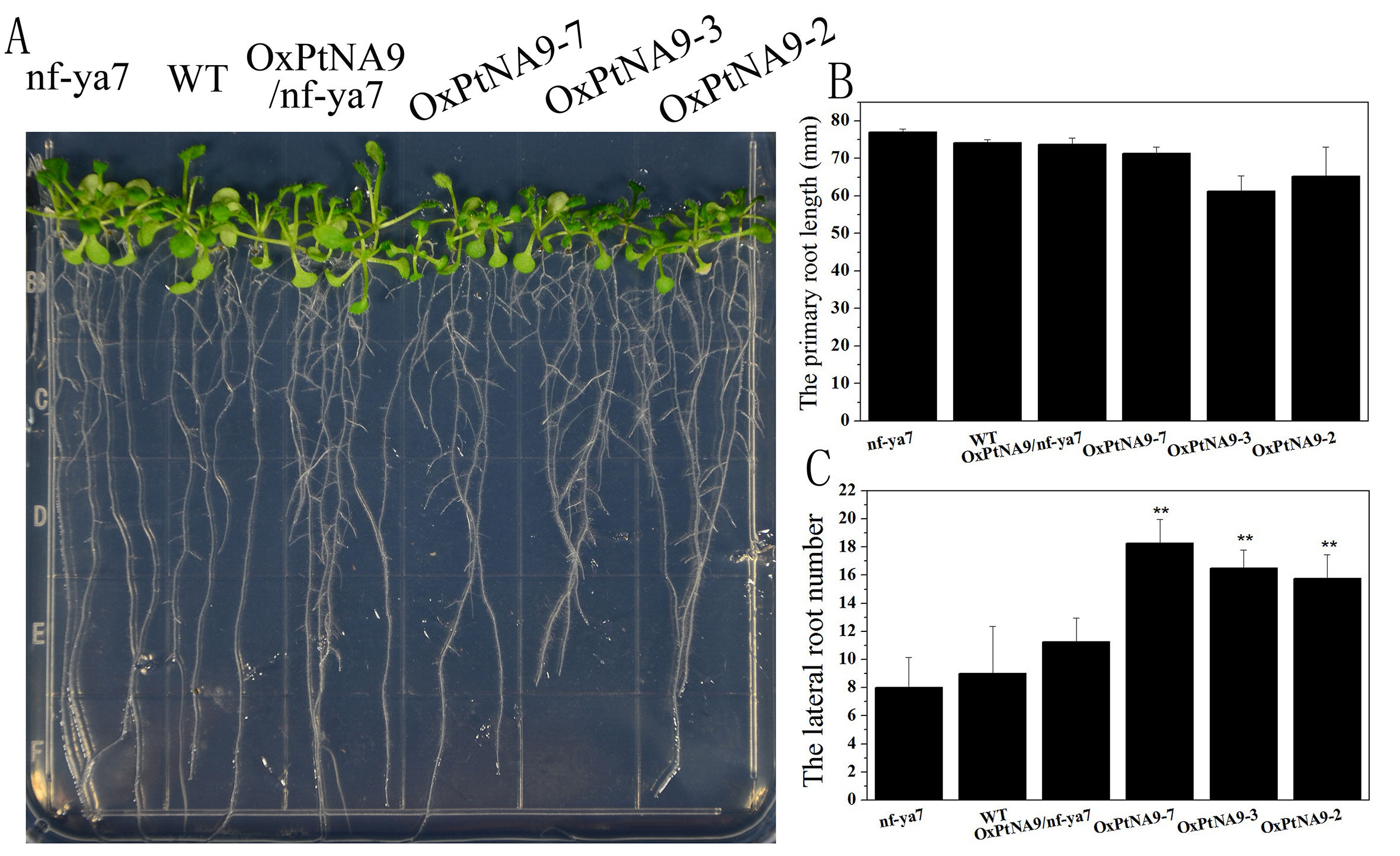

Supplement: FIGURE S4 — Overexpression of PtNF-YA9 grown under 1/2 MS agar medium at seedling stage in Arabidopsis. (A) Morphological differences of root phenotypes in 1/2 MS agar medium plates. (B) Primary root length calculated at different lines. (C) Lateral root numbers calculated at different lines. [file Image_4.JPEG]

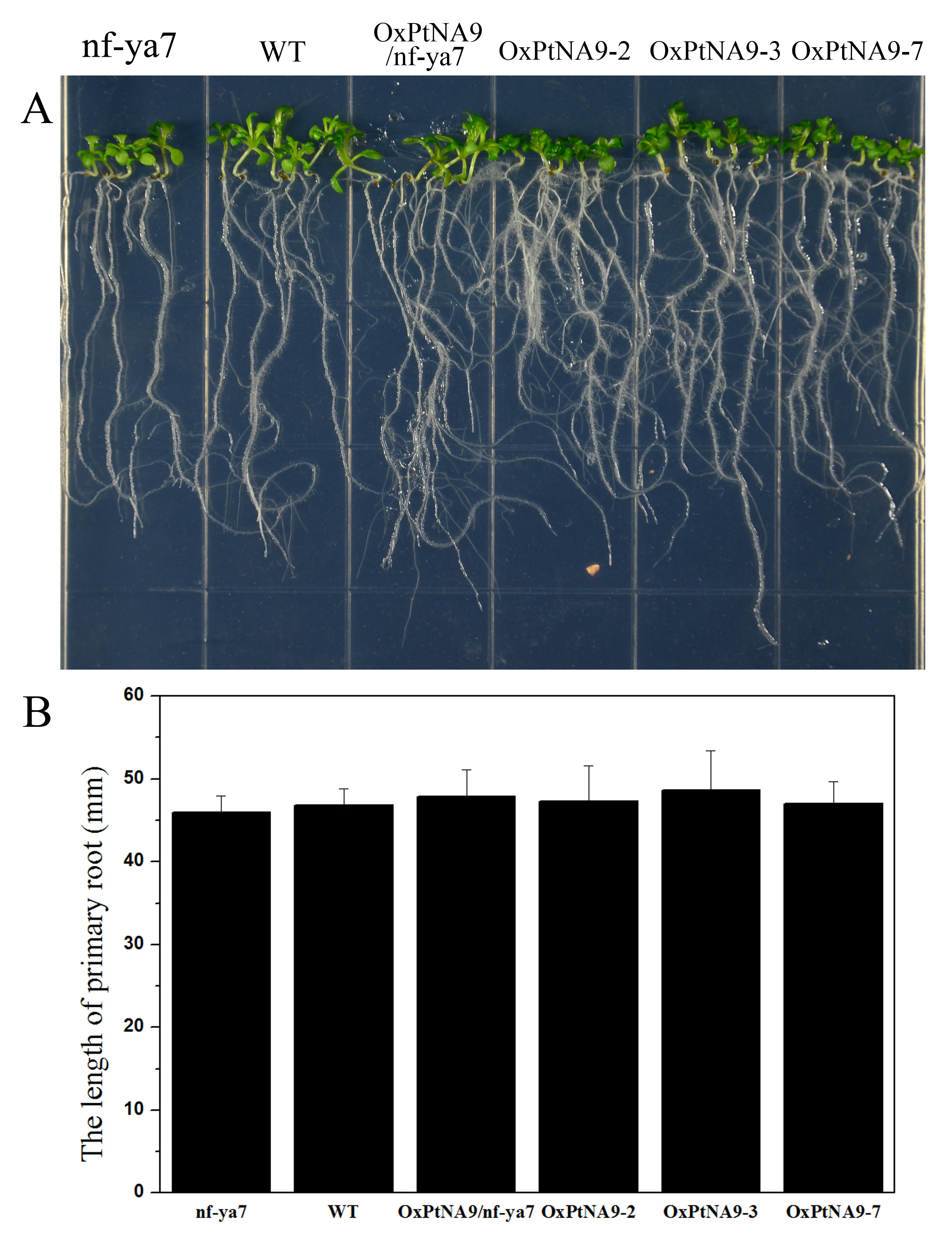

Supplement: FIGURE S5 — Overexpression of PtNF-YA9 confers drought tolerance at seedling stage in Arabidopsis. (A) Morphological differences in 1/2 MS agar medium plates with 200 mM mannitol. (B) Primary root length of different plants calculated under treatment of 200 mM mannitol. [file Image_5.JPEG]

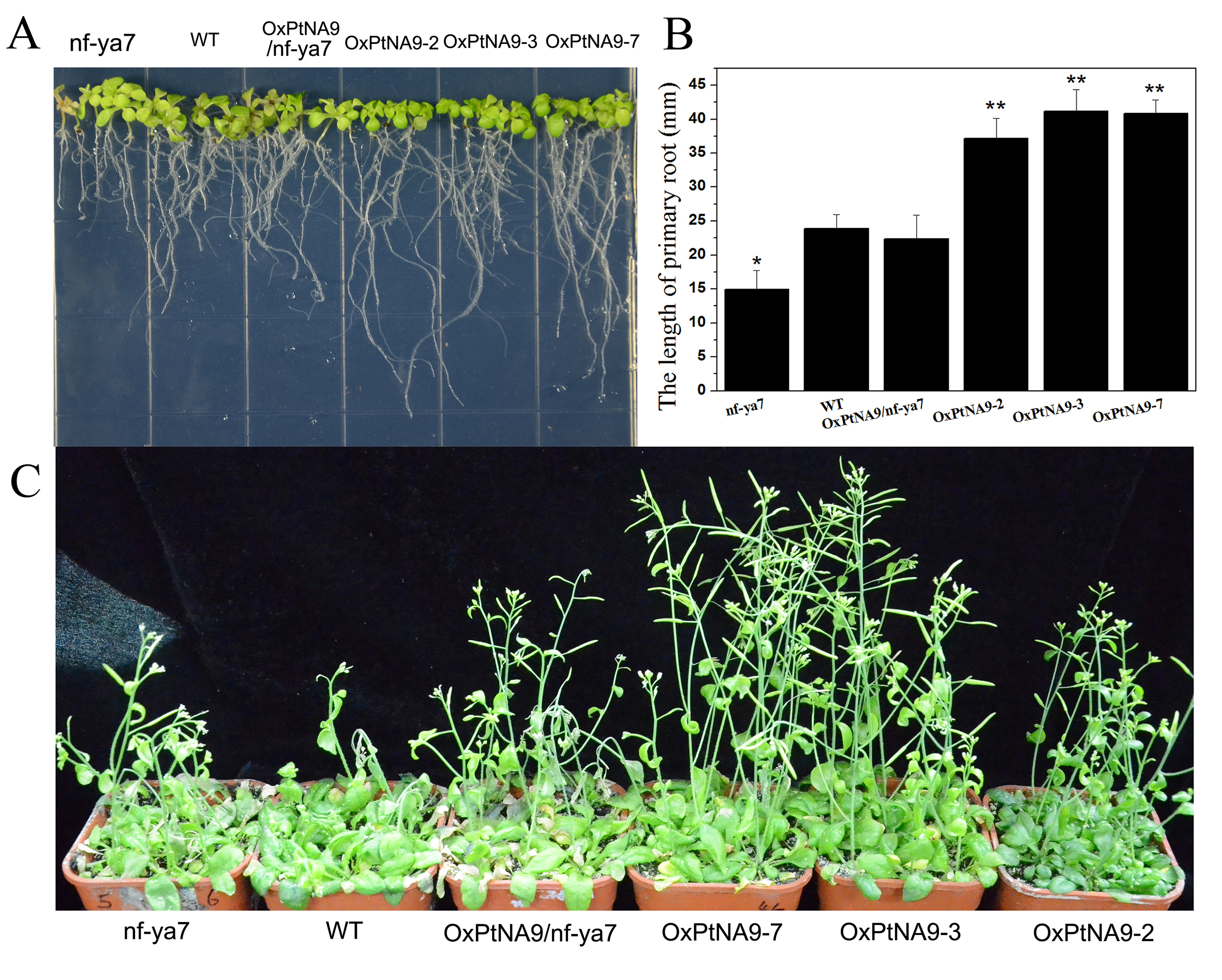

Supplement: FIGURE S6 — PtNF-YA9 overexpression confers salt tolerance in Arabidopsis. (A) Morphological differences in 1/2 MS agar medium plates with 100 mM NaCl. (B) Primary root length of different plants calculated under treatment of 100 mM NaCl. (C) Morphological differences in long-term salt treatment. The seedlings were grown in soil for 2 weeks under well-watered conditions and thereafter subjected to salt stress by pouring 100 mM NaCl–water every 5 days until 4 weeks. [file Image_6.JPEG]
