# Supplementary material for: Populus trichocarpa PtNF-YA9, A Multifunctional Transcription Factor, Regulates Seed Germination, Abiotic Stress, Plant Growth and Development in Arabidopsis
Source: Front Plant Sci. 2018 Jul 9;9:954. doi: 10.3389/fpls.2018.00954 (PMC6052803; doi:10.3389/fpls.2018.00954)
Supplement: TABLE S1 — Primers used in this research. [file Table_1.DOC]

| **Table S1. Primers used in this research.** | | |
| --- | --- | --- |
| **Primers Name** | **primers sequence** | **Primers used** |
| NA9-F | CCTCATGACTTCTTCGGTGCAT | For PtNF-YA9 gene cloning |
| NA9-R | CCATCACTTCCAGTGTATCAAGACT |
| NA9-qF | TCAAGTCTCGGAAGCCATACT | For PtNF-YA9 gene qRT-PCR |
| NA9-qR | TTGTCATCCAAGGAAGCAAT |
| NA9-Fz | AACACGGGGGACTCTTGACCATGACTTCTTCGGTGCATGATC | For transformation of Arabidopsis vector construct |
| NA9-Rz: | GATCGGGGAAATTCGAGCTGTCAAGACTTGTCATCCAAGGAAG |
| NA9-GFP-Fz | AACACGGGGGACTCTTGACCATGACTTCTTCGGTGCATGATC | For Subcellular localization vector construction |
| NA9-GFP-Rz | CCTTTACTAGTCAGATCTACAGACTTGTCATCCAAGGAAG |
| NA9Pro-F | GCCAGGTATGGTGAAAACGGAAT | For PtNF-YA9 promoter cloning |
| NA9Pro-R | CCAGAAAGATCATGCACCGAAG |
| NA9pz-F | TCGAGCTCGGTACCCGGGGCCAGGTATGGTGAAAACG | PtNF-YA9 promoter vector construction |
| NA9pz-R | CTTTACTAGTCAGATCTACCATGAGGCTGTTATGTCCACAG |
| GAPDH | ACCACTGTCCACTCTATCACTGC | qPCR of internal control gene |
| GAPDH | TGAGGGATGGCAACACTTTCCC |
| ACT2_F | TATGAATTACCCGATGGGCAAG | qPCR of internal control gene |
| ACT2_R | TGGAACAAGACTTCTGGGCAT |
| ABI5-F | CGGAGACAGAACGAGGGAAAA | qPCR of stress related genes |
| ABI5-R | TCCTCCTACCAACACAGAAAACA |
| DREB2A_F | GACCTAAATGGCGACGATGT | qPCR of stress related genes |
| DREB2A_R | TCGAGCTGAAACGGAGGTAT |
| DREB2B_F | TTGCGACTATAAAGAAGAAG | qPCR of stress related genes |
| DREB2B_R | TCCGCGGTAGGAAAAGTACC |
| RD29A_F | GATATCGACAAGGATGTGCCG | qPCR of stress related genes |
| RD29A_R | GTATCCAGGTCTTCCCTTCGC |
| RD29B_F | TTCTGACCACACCAAACCCAT | qPCR of stress related genes |
| RD29B_R | CAGCCAGTGCCTCATGTCC |
| ABF1-F | TCCGTGGTAAGAAGGTGAAGTC | qPCR of stress related genes |
| ABF1-R | TGTAAGCGTCTCTGTAATTTCTCC |
